# Determinants of Work Performance in Workers with Depression and Anxiety: A Cross-Sectional Study

**DOI:** 10.3390/ijerph14050466

**Published:** 2017-04-26

**Authors:** Ivana Ivandic, Kaloyan Kamenov, Diego Rojas, Gloria Cerón, Dennis Nowak, Carla Sabariego

**Affiliations:** 1 Department of Medical Informatics, Biometry and Epidemiology–IBE, Chair for Public Health and Health Services Research, Research Unit for Biopsychosocial Health, Ludwig-Maximilians-Universität (LMU), Marchioninistr. 17, 81377 Munich, Germany; Carla.Sabariego@med.uni-muenchen.de; 2 Instituto de Salud Carlos III, Centro de Investigación Biomédica en Red de Salud Mental (CIBERSAM), Diego de León 62, 28006 Madrid, Spain; kaloyan.kamenov@cibersam.es; 3 Departamento de Estudios, Servicio Nacional de la Discapacidad (Senadis), Ministerio de Desarrollo Social, Catedral 1575, 8340309 Santiago, Chile; diego.rojasn@gmail.com (D.R.); gceron@senadis.cl (G.C.); 4 Institute and Outpatient Clinic for Occupational, Social and Environmental Medicine, Ludwig-Maximilians-Universität (LMU), Ziemssenstr. 1, 80336 Munich, Germany; Dennis.Nowak@med.uni-muenchen.de

**Keywords:** mental health, anxiety, depression, workplace, work performance, environmental factors, disability

## Abstract

Depression and anxiety are highly prevalent disorders with an impact on existential aspects of person’s life, including employment i.e., work performance (WP). In order to develop appropriate strategies, it is essential to identify determinants of WP. The objective of this study was to identify the built, social, attitudinal and health system-related environmental determinants of WP in workers with anxiety or depression in total (*N* = 1211) and regarding the level of disability. Hierarchical binary logistic regression was performed on data obtained from implementation of the WHO Model Disability Survey (MDS) in Chile in 2015. Hindering aspects of means of transportation and workplace, and the use of personal assistance were determinants of WP for all workers with anxiety or depression. Results differed with level of disability. Hindering aspects of means of transportation and workplace, and discrimination were determinants of WP for persons with mild to moderate disability, while hindering aspects of the workplace and dwelling, and the use of personal assistance were determinants of WP for persons with severe disability. Our results emphasize the need for a broader understanding of determinants of WP and the requirement for an integrative approach in developing both universal and specific strategies that go beyond workplace settings.

## 1. Introduction

Depression and anxiety are highly prevalent groups of mental disorders that are costly and significant contributors to the global burden of disease. Estimates on the lifetime prevalence of depression and anxiety range between 4–16.6% [1,2,3] and 3.8–25% [4], respectively. According to the Global Burden of Disease Study (GBD), depressive disorders were the third and anxiety the ninth leading cause of global disability in 2015 [5]. They are also associated with the highest productivity-loss related costs of all chronic illnesses [6]. The total costs of depression in the European Union (EU) have been estimated at €118 billion per year, of which 64% are due to productivity losses [7]. Additionally, the average annual costs, including medical, pharmaceutical and disability costs for workers with depression has been reported to be 4.2 times higher than those incurred by the usual beneficiary [8].

The individual burden of depression and anxiety impacts existential aspects of an individual’s life, including employment. Compared to the general population, persons with mental disorders, including depression and anxiety, experience on average 15–30% lower employment rates, and long-term unemployment [7] can be twice as high [9]. For those who succeed in obtaining employment, there is an increased risk of exposure to inequalities at work, such as lower salaries and discrimination [10]. Persons with depression and anxiety also have increased absenteeism and presenteeism rates, as well as low productivity [11] resulting from decreased work performance [6]. Recent research linked depression to reduced work participation (e.g., time to return to work and work status) [12] and common mental disorders, including both depression and anxiety, to problems in work performance [12,13].

Work performance (WP) is a multidimensional construct that includes a worker’s experience in fulfilling their work tasks and “results from the relationship between an individual’s health resources and the expectations and structural conditions that operate within social settings such as the workplace” [12]. Evidence shows that workers with depression can only achieve an acceptable WP with extra effort, reporting on average 11.6 days requiring extra effort to be productive in the previous month, while workers with anxiety experience significantly more frequent days of partial inability to function normally at work [14]. In addition, workers with depression and anxiety have two and almost six times higher risk, respectively, of experiencing problems in WP in comparison to other workers [6]. Hence, effective strategies in terms of adaptations and accommodations, that would enable workers with depression and anxiety to achieve WP levels comparable to the general population, are warranted.

The Convention on the Rights of Persons with Disabilities (CRPD) provides a foundation that protects the rights of persons with disabilities (PwD), including persons with mental disorders. According to the Convention, PwD include “those who have long-term physical, mental, intellectual or sensory impairments which in interaction with various barriers may hinder their full and effective participation in society on an equal basis with others” [15]. Barriers PwD encounter refer not only to the physical but also attitudinal or social environment that can act in a hindering or facilitating way. Article 27 of the CRPD provides a legal framework for the right of PwD to work on an equal basis with others. Among other things, it considers prohibition of discrimination with regard to access to the labor market and open, inclusive and accessible work environments [15]. To meet the CRPD mandate, ratified countries must ensure that effective strategies targeting barriers hindering optimal WP are in place. There is a substantial body of evidence that an individual’s work environment, specifically psychosocial risk factors such as high job demands, low control or social support, are risk factors associated with depression and anxiety [16]. However, existing strategies mainly focus on the individual level, i.e., symptom reduction [16,17,18,19]. Evidence on the effectiveness of organizational strategies addressing work environment, adaptations and accommodations is scarce.

Although appropriate organizational strategies can only be effective if targets are clearly defined, evidence on which barriers are the most hindering for persons with depression and anxiety is still scarce. Recent studies have mostly addressed the relationship between work performance and personal factors (e.g., sociodemographic factors or personality traits) [20,21], work-related factors (e.g., employment characteristics, types of company, supervisory behavior) [22] and disorder-related factors (e.g., symptom severity, clinical history) [23,24,25,26,27,28,29,30]. However, there is a gap in knowledge of which built, political, social and attitudinal environmental factors (EF) might impact WP of persons with depression and anxiety. Due to the complexity and developmental nature of WP, a broadening of determinants of WP has been called for in a recent systematic review [31].

The main objective of this study is to identify the built, social, attitudinal and health system-related environmental determinants of WP in workers with anxiety or depression, for the total sample and for subgroups taking into account level of disability. This study uses data from a national implementation of the Model Disability Survey (MDS), developed by the World Health Organization (WHO) and World Bank [32]. The MDS is grounded in the biopsychosocial International Classification of Functioning, Disability and Health (ICF) model [33] and measures performance in different life domains, including work, as well as a broad range of EF such as social support, discrimination, accessibility to treatment, accommodations at work and accessibility to aids and devices. This study will provide valuable information for policy makers on how to design future appropriate strategies to improve WP in persons with anxiety or depression. In addition, this study conforms to one of the major requirements of the 2030 Agenda for Sustainable Development set by the United Nations (UN) to reach the Sustainable Development Goals (SDG) [34], namely disaggregation by disability.

## 2. Materials and Methods

### 2.1. Study Design and Participants

We conducted secondary data analysis of the implementation of the MDS as a national disability survey in Chile (ENDISC II) carried out in 2015, using a large representative sample of the general population including more than 17,000 individual interviews. Participants of the survey were children and adults from 15 provinces. ENDISC II is based on the MDS, a project (http://www.who.int/disabilities/data/mds.pdf) initiated by the WHO and the World Bank in 2011. In the MDS, disability is conceptualized as the outcome of interactions between a person with a health condition and various environmental and personal factors. The survey utilizes a general population sample without screeners or filters and enables a direct comparison between groups with differing levels and profiles of disability, including a comparison to persons without disability. The current MDS Alpha version questionnaire consists of eight modules, and the ones implemented in Chile were module 3000: environmental factors; module 4000: functioning; and module 5000: health conditions and capacity. Additionally, the ENDISC II collected information on sociodemographic characteristics, work and health care resources utilization.

Employed persons with anxiety or depression of a working age (18–64 years old) were included in the present study (*N* = 1211). Persons were considered employed if, in the previous week, they worked at least one hour in a productive activity (excluding housework) for pay in cash or any kind, or if they worked on a casual or occasional activity (e.g., one-time occasion or part-time work). The presence of anxiety or depression was assessed based on the Self-Administered Comorbidity Questionnaire (SCQ) [35]. This questionnaire includes a list of country-specific high prevalent or high priority health conditions and for each condition participants were asked, “Do you have [DISEASE NAME]?” Comorbidities between depression and anxiety were allowed for.

### 2.2. Variables

#### 2.2.1. Dependent Variable

WP was the dependent variable, operationalized with the question from module 4000, “How much of a problem is getting things done as required at work?” Responses were recorded on a 5-point Likert scale ranging from 1 (“none”) to 5 (“extreme”). For the purpose of this study, the variable was dichotomized into “no problems” (response category 1) and “problems” (response categories 2–5). It is important to note that, while answering in module 4000, respondents are requested to take into account both health problems and EF.

#### 2.2.2. Independent Variables

The following EF of module 3000 were included: hindering or facilitating aspects of health facilities, places to socialize, workplace, shops or banks, places to worship, transportation, dwelling, terrain and climate, lighting, noise and crowds. For each of these, respondents were asked to what extend these aspects of the general environment make it easy or hard for them to do what they want or need to do. The original responses were recorded on a 5-point Likert scale ranging from 1 (“very easy”) to 5 (“very hard”). These variables were recoded into three categories: “facilitating” (response categories 1–2), “neutral” (response category 3) and “hindering” (response categories 4–5). Use of personal assistance and assistive devices and modifications were dichotomous variables (“use” and “do not use”). Use of health care services and rehabilitation service in the last 12 months were included as dichotomous variables as well (“yes” and “no”). Perception of discrimination in the last 12 months was expressed on a 5-point Likert scale ranging from 1 (“not discriminated”) to 5 (“extremely discriminated”). This variable was dichotomized into “no” (response category 1) and “yes” (response categories 2–5).

#### 2.2.3. Control Variables

The following control variables were considered: sex, age, education, marital status and level of disability operationalized as capacity difficulties. Capacity refers to the health state of the individual considering the impact of one or more health conditions. The capacity variable is metrical, ranges from 0 (“no difficulties”) to 100 (“extreme difficulties”), and was previously estimated by the National Disability Service of Chile using partial credit model analysis. In this study, levels capacity was stratified using cut-off points previously defined by the WHO [32]. Capacity scores >44.1 pointed out severe difficulties in capacity, scores between 30 and 44.1 denoted moderate difficulties, and scores <30 denoted mild or no difficulties. Capacity was considered to be a strong potential confounder in this study as it has an important impact on the performance in daily life and at work. Therefore, we adjusted the regression model targeting the complete sample for capacity. Additionally, stratified analysis by level of difficulties in capacity was carried out.

### 2.3. Statistical Analysis

Hierarchical binary logistic regression was performed to identify environmental determinants of WP for the total sample of workers with anxiety or depression, and stratified by levels of difficulties in capacity. Due to the relatively small sample size, the mild and moderate groups were merged and compared to the group with severe difficulties. Analyses were adjusted for sex, age, education and marital status and, in the case of the whole sample, also for capacity level. For each factor, Wald statistics was estimated and factors showing significant association (*p* < 0.05) with WP were selected for the final model. Hosmer-Lemeshow test and statistic were considered for overall model goodness-of-fit. Percentage of correctly classified cases was used as the model’s predictive power measure. In addition, Nagelkerke’s R2 was considered as a measure of variance explained by the model. Odds ratios (OR) and their 95% confidence intervals (95% CI) were calculated. The number of missing values in the factors was at most 2.0%, therefore no imputation was used. The final models were selected on the principle of parsimony. Data were analysed using IBM SPSS Statistics for Windows, version 24 (IBM Corp., Armonk, NY, USA).

## 3. Results

Participants were predominantly male (65.5%) with a mean age of 43.20 years (standard deviation (SD) = 11.81). The majority had anxiety (41.1%), while depression and both depression and anxiety (comorbidity) were reported by 31.5% and 27.4% of respondents, respectively. Most participants did not experience discrimination (74.1%). Characteristics of participants are presented in Table 1.

### 3.1. Total Sample (N = 1211)

The final logistic regression model included 1155 individuals, and correctly classified 83.4% of cases and explained 31.5% of the variance in WP. The Hosmer-Lemeshow test indicated a good model fit (χ^2^ = 7.256, *p* = 0.509, *df* = 8). Hindering and facilitating aspects of means of transportation and workplace as well as the use of personal assistance were significant determinants of WP. In comparison to individuals who considered the transportation facilitating, those who considered it as hindering had almost two times higher risk of experiencing problems in WP (OR = 1.977; 95% CI = 1.358–2.878). Workers who experienced their workplace as hindering had about 4.5 higher risk (OR = 4.498; 95% CI = 2.866–7.062) of having problems in WP, while this risk was still approximately 2.5 higher for individuals who perceived their workplace as neutral (OR = 2.513; 95% CI = 1.575–4.009). Regarding the use of personal assistance, the risk of problems in WP was more than twice as high for workers who used personal assistance than for those who did not (OR = 2.327; 95% CI = 1.410–3.841). No interaction effects were found. Results are presented in Table 2.

### 3.2. Group with Mild to Moderate Levels of Difficulties in Capacity (N = 733)

The logistic regression model, adjusted for sex, age, education and marital status, included 733 participants and correctly classified 90.7% of cases, explained 13.2% of the variance in WP, and had a good fit (χ^2^ = 5.062, *p* = 0.751, *df* = 8). Hindering and facilitating aspects of means of transportation and workplace as well as discrimination were significant determinants of WP. Workers who experienced their transportation and workplace as hindering had about 3.2 (OR = 3.118; 95% CI = 1.737–5.597) and 3.5 (OR = 3.481; 95% CI = 1.704–7.112) times higher risk of having problems in WP. The risk was even higher regarding perception of discrimination; workers who felt discriminated were almost as twice as likely to have problems in WP (OR = 1.877; 95% CI = 1.064–3.312). Differing from the results for the total sample, the use of personal assistance was not a significant determinant for this group. No interaction effects were found. Results are presented in Table 2.

### 3.3. Group with Severe Levels of Difficulties in Capacity (N = 429)

The logistic regression, adjusted for sex, age, education and marital status, included 429 participants and correctly classified 73.2% cases, explained 23.2% of the variance, and had a good fit (χ^2^ = 5.984, *p* = 0.649, *df* = 8). Hindering and facilitating aspects of the workplace and dwelling as well as personal assistance were significant determinants of WP. Compared to individuals who had a facilitating workplace, those whose workplace was hindering had an almost 6 times greater risk of experiencing problems in WP (OR = 5.791; 95% CI = 3.169–10.583). This risk was almost 3 times higher for individuals who were neutral regarding their workplace, in comparison to those who considered their workplace facilitating (OR = 2.747; 95% CI = 1.431–5.271). Individuals who used personal assistance had a 2.3 higher risk of experiencing problems in WP, in comparison to those who did not use personal assistance (OR = 2.333; 95% CI = 1.337–4.070). Workers experiencing a hindering dwelling had more than 2 times higher risk of having problems in WP (OR = 2.201; 95% CI = 1.190–4.073), compared to workers whose dwelling was facilitating. Neither transportation, perceived discrimination nor any other EF were significant determinants for this group, and no interaction effects were found. Results are presented in Table 2.

## 4. Discussion

The objective of the current study was to identify the built, social, attitudinal and health system-related related environmental determinants of work performance in workers with anxiety or depression in general, and taking into account disability, operationalized as levels of difficulties in capacity. Hindering aspects of transportation and workplace as well as use of personal assistance are determinants of WP for all workers with anxiety or depression. Results differ, however, when the level of disability is taken into account. Hindering aspects of transportation and the workplace as well as discrimination are determinants of WP for workers with mild and moderate disability levels, while hindering aspects of the workplace and dwelling, and the use of personal assistance are determinants of WP for persons with severe disability. Disaggregation by disability is one of the major requirements to reach the SDGs [34], and our study corroborates its relevance. By disaggregating the sample, we learn that persons with different disability levels experience either the same barrier but to different extents (i.e., hindering workplace) or different barriers (i.e., hindering transportation and dwelling, discrimination and use of personal assistance). As a consequence, policy makers and other stakeholders must target both universal and specific strategies to effectively improve WP.

Similar to previous research, we found a hindering workplace to be a determinant of problems in WP. A growing body of evidence suggests a strong association between work conditions and performance in the general population [36,37,38] and our results corroborated this association in workers with anxiety or depression. While our study clearly shows the negative impact of hindering or even neutral aspects of the workplace, it lacks information on which specific workplace factors hinder WP. Previous studies, identified in a recent systematic review [39], focused on factors impacting return to work and work limitations in persons with mental disorders and highlighted the importance of changing work tasks and supervisor communication with employees. Considering the paucity of research on workplace factors predictive of WP, further studies are needed to identify more specific factors.

An even more important finding of our study is, however, the identification of environmental factors beyond an individual’s workplace as determinants of WP. Personal assistance is a determinant both for all workers with depression or anxiety, and for the strata with severe disability; persons using personal assistance are about twice as likely to experience problems in WP as persons who have no assistance. This might sound intriguing at a first glance, but a potential explanation is that persons with severe disability are those who are in need and entitled to receive personal assistance. Another potential explanation could be the high levels of comorbidity between depression or anxiety and other rather “physical” conditions. For example, evidence show that the prevalence of depression is increased in cardiovascular disease, and up to 40% of people have either major or minor depression following a myocardial infarction [40,41]. Hindering aspects of means of transportation is another non-workplace determinant of problems in WP for all workers and especially for persons with mild and moderate disability. The identification of personal assistance and transportation as relevant determinants is in line with a study ranking and comparing EF most responsible for the disability experienced by persons with mental disorders and persons with four further major non-communicable conditions [42]. Finally, hindering aspects of the dwelling is an important determinant of WP for persons with anxiety or depression with severe disability. Similarly, dwelling has been identified as a relevant EF impacting the overall performance of persons with severe level of disability [43]. A key lesson learned from our study is that a person’s life should not be strictly divided into “private” and “work” spheres, but rather considered from a holistic perspective since strategies targeting, for instance, the accessibility of transportation might also have an impact on WP. Given the importance of non-workplace EF, integrated approaches and cross-cutting strategies that go beyond symptoms and aspects of the workplace are needed when developing strategies to improve WP of workers with anxiety or depression.

Our work meets the current calls for complementing previous research on determinants of WP among workers with anxiety or depression [12]. Previous studies, included in the systematic review of Lagerveld et al. [12], identified mainly disorder-related (e.g., severity of symptoms), personal (e.g., gender, personality traits), and work-related (e.g., type of occupation) predictors of WP in persons with depression. This review concluded that, considering the complexity and developmental nature of WP, a broadening of the concept would be needed in future studies. Our study meets this call by addressing environmental determinants of WP from a broader perspective, including aspects of the general environment, personal assistance or use of health services. This was possible because we used data from a general population survey targeting functioning and disability and not from a labor or a workplace risk assessment survey, as is usually conducted in the field of research on work. However, our regression models explain a small proportion of variance, suggesting that information on specific aspects of the workplace such as job type and content, workload or organizational culture—usually included in research on work—would have been important too. We conclude that the broadening of the WP concept and its determinants also requires a broadening of the type of data about EF included in labor or workplace risk assessment surveys. This would allow for holistic data analyses strategies that take into account both general EF and specific workplace factors.

An interesting finding of our study is that discrimination is a significant determinant of WP only for workers with anxiety or depression with mild to moderate disability. This is surprising, since discrimination and stigma are commonly experienced by persons with mental disorders generally [44,45] as well as in the workplace [10]. It is acknowledged that discrimination is associated with cultural context as well as with the age and gender of the individual [45]. Our study clearly demonstrates an important association of discrimination with the experienced level of disability, pointing out persons with rather low levels of disability as the target group for specific strategies.

This study reinforces the need of going beyond diagnosis and disaggregating data by levels of disability. Our results demonstrate that workers with anxiety or depression experience either the same barrier but to different extents (i.e., hindering workplace) or different barriers (i.e., hindering transportation, dwelling and discrimination), implying the need for both universal and specific strategies. Taking disability into account is in line with the requirements of the CRPD and of the SDG [34], and our study presents an exemplary work on how disaggregation can be done using data from an ICF-based disability survey.

Some limitations of this study need to be considered. Information on specific aspects of the workplace such as job type and content, workload or organizational culture should have been added to the regression models. However, these variables were not available in the kind of data used, a disability survey. Nevertheless, our study provides relevant information about variables not generally included in labor or demographic surveys. Due to the relatively small sample size of workers with depression or anxiety across categories, we had to create a dichotomous WP variable (“problems” versus “no problems”) and were not able to identify determinants of mild, moderate or severe problems in WP. In addition, WP was assessed with only one self-report question, although WP should usually be assessed with specific questionnaires. This study used a self-report questionnaire of health conditions and has the risk of overestimating the number of persons with anxiety or depression. In contrast, there is a possibility of underestimating this number by using common diagnostic criteria. Since cognitive distortion can be present in persons with depression and anxiety, the assessment of WP and the environmental factors could be biased. Finally, we have data from one country, Chile, which limits the generalizability of results. Studies including additional countries are needed to confirm our results.

## 5. Conclusions

Environmental factors within and outside of the workplace are important determinants of WP among workers with depression or anxiety, emphasizing the need for an integrative approach in developing strategies that go beyond the workplace setting. Hindering aspects of transportation and the workplace as well as use of personal assistance are determinants of WP for the total sample. When the sample is disaggregated by disability level, hindering aspects of transportation and workplace as well as discrimination are significant determinants of WP for persons with mild to moderate disability levels, while hindering aspects of the dwelling, workplace, and use of personal assistance are significant determinants of WP for persons with severe disability. Since persons with different levels of disability experience either the same barrier but to different extents (i.e., hindering workplace) or different barriers (i.e., hindering transportation, dwelling and discrimination), both universal and specific strategies are needed. This study shows the importance of using a broader understanding of determinants and filling in the gap in knowledge of which built, political, social and attitudinal EF might impact WP of persons with depression and anxiety.

## Figures and Tables

**Table 1:** Characteristics of workers with anxiety or depression in the total sample and in the subgroups with and without problems in work performance (WP).

Variable	Category	Total Sample		Subsample with No Problems in WP **		Subsample with Problems in WP **	
		*N*	%	N	%	N	%
		**1211**	**100**	**954**	**78.8**	**238**	**19.7**
Sex	Male	793	65.5	619	64.9	166	69.7
	Female	418	34.5	335	35.1	72	30.3
Education	None/Elementary	248	20.5	173	18.1	71	29.8
	Secondary	575	47.5	460	48.2	105	44.1
	Tertiary	388	32.0	321	33.6	62	26.1
Marital status	Single	396	32.7	315	33.0	77	32.4
	Married/living together	562	46.4	448	47.0	103	43.3
	Separated/divorced	217	17.9	165	17.3	49	20.6
	Widowed	36	3.0	26	2.7	9	3.8
Self-reported mental disorder	Depression	381	31.5	531	30.4	170	36.1
	Anxiety	498	41.1	664	44.3	152	28.6
	Depression and anxiety	332	27.4	241	25.3	84	35.3
Perceived discrimination	No	897	74.1	727	76.2	157	66.0
	Yes	314	25.9	227	23.8	81	34.0
Capacity level *	Mild level of difficulties	377	31.1	351	36.8	22	9.2
	Moderate level of difficulties	390	32.2	333	34.9	52	21.8
	Severe level of difficulties	443	36.6	269	28.2	164	68.9

* Data was obtained on *N* = 1210 participants, capacity score not available for one person; ** data was obtained on *N* = 1192 participants, WP score was not available for 19 persons.

**Table 2:** Binary logistic regression models for the total sample (*N* = 1155) and the strata by level of difficulties in capacity. Odds ratios (OR) and 95% confidence intervals (95% CI) predicting the risk of experiencing problems in work performance (WP) for workers with anxiety or depression are reported. Variables that did not remain in the regression models, because they were not statistically significant, are identified as “not included”.

Variable (Reference Group)	Total Sample (*N* = 1155)	Mild and Moderate Difficulties in Capacity (*N* = 733)	Severe Difficulties in Capacity (*N* = 429)
	OR (95% CI)	OR (95% CI)	OR (95% CI)
Control variables			
Capacity (mild levels of difficulties)			
Moderate level of difficulties	2.397 (1.370–4.193) *		
Severe level of difficulties	8.293 (4.894–14.052) *		
Sex (female)	1.020 (0.701–1.482)	1.156 (0.659–2.030)	0.958 (0.577–1.591)
Age	0.971 (0.955–0.987) *	0.962 (0.936–0.988) *	0.979 (0.959–1.000) *
Education level (No education/Elementary)			
Secondary	0.578 (0.381–0.879) *	0.549 (0.268–1.127)	0.618 (0.366–1.044)
Tertiary	0.533 (0.330–0.862) *	0.464 (0.213–1.010)	0.627 (0.333–1.180)
Marital status (Single)			
Married/living together	1.019 (0.681–1.525)	0.909 (0.487–1.695)	1.157 (0.678–1.975)
Separated/divorced	1.311 (0.792–2.169)	1.516 (0.650–3.538)	1.340 (0.717–2.501)
Widowed	2.444 (0.909–6.570)	2.079 (0.406–10.634)	2.549 (0.704–9.226)
Environmental predictors			
Workplace (Facilitating)			
Neutral	2.513 (1.575–4.009) *	1.591 (0.734–3.447)	2.747 (1.431–5.271) *
Hindering	4.498 (2.866–7.062) *	3.481 (1.704–7.112) *	5.791 (3.169–10.583) *
Transportation (Facilitating)			*not included*
Neutral	1.390 (0.834–2.316)	1.221 (0.538–2.772)	
Hindering	1.977 (1.358–2.878) *	3.118 (1.737–5.597) *	
Dwelling (Facilitating)	*not included*	*not included*	
Neutral			1.323 (0.652–2.684)
Hindering			2.201 (1.190–4.073) *
Use of personal assistance (Do not use)		*not included*	
Use	2.327 (1.410–3.841) *		2.333 (1.337–4.070) *
Discrimination (Not discriminated)	*not included*		*not included*
Discriminated		1.877 (1.064–3.312) *	

* *p* < 0.05.
